# Process Parameter Optimization of a Polymer Derived Ceramic Coatings for Producing Ultra-High Gas Barrier

**DOI:** 10.3390/ma14227000

**Published:** 2021-11-18

**Authors:** Iftikhar Ahmed Channa, Aqeel Ahmed Shah, Muhammad Rizwan, Muhammad Atif Makhdoom, Ali Dad Chandio, Muhammad Ali Shar, Asif Mahmood

**Affiliations:** 1 Department of Metallurgical Engineering, NED University of Engineering and Technology, Off University Road, Karachi 75270, Pakistan; aqeelshah@neduet.edu.pk (A.A.S.); engr.rizwan@neduet.edu.pk (M.R.); alidad@neduet.edu.pk (A.D.C.); 2 Institute of Metallurgy and Materials Engineering, University of the Punjab, Lahore 54590, Pakistan; atif.imme@pu.edu.pk; 3 Department of Mechanical & Energy Systems Engineering, Faculty of Engineering and Informatics, University of Bradford, Bradford BD7 1DP, UK; m.baloch@bradford.ac.uk; 4 Chemical Engineering Department, College of Engineering, King Saud University Riyadh, Riyadh 11451, Saudi Arabia

**Keywords:** silica coatings, polysilazane, process optimization, thin films, room temperature cured PHPS, oxygen and moisture permeability

## Abstract

Silica is one of the most efficient gas barrier materials, and hence is widely used as an encapsulating material for electronic devices. In general, the processing of silica is carried out at high temperatures, i.e., around 1000 °C. Recently, processing of silica has been carried out from a polymer called Perhydropolysilazane (PHPS). The PHPS reacts with environmental moisture or oxygen and yields pure silica. This material has attracted many researchers and has been widely used in many applications such as encapsulation of organic light-emitting diodes (OLED) displays, semiconductor industries, and organic solar cells. In this paper, we have demonstrated the process optimization of the conversion of the PHPS into silica in terms of curing methods as well as curing the environment. Various curing methods including exposure to dry heat, damp heat, deep UV, and their combination under different environments were used to cure PHPS. FTIR analysis suggested that the quickest conversion method is the irradiation of PHPS with deep UV and simultaneous heating at 100 °C. Curing with this method yields a water permeation rate of 10^−3^ g/(m^2^⋅day) and oxygen permeation rate of less than 10^−1^ cm^3^/(m^2^·day·bar). Rapid curing at low-temperature processing along with barrier properties makes PHPS an ideal encapsulating material for organic solar cell devices and a variety of similar applications.

## 1. Introduction

Many applications such as: solar cells [1] light-emitting diodes (LEDs) [2], organic transistors [3], and photodetectors [4] require encapsulating materials that should not only be transparent, but also an excellent barrier against the diffusion of oxygen and moisture at the same time [5,6,7,8,9,10]. Therefore, vacuum evaporated silica coatings have widely been used for this purpose [11]. Evaporated coatings are usually produced by methods including physical vapor deposition (PVD), chemical vapor deposition (CVD), plasma enhanced chemical vapor deposition (PECDV), magnetron sputtering and atomic layer deposition (ALD), etc. [6,11,12,13]. These techniques are reported in the literature to produce ultra-high oxygen and moisture diffusion barriers (i.e., down to 10^−6^ g/m^2^·day) for the protection of OLEDs [6,14]. These techniques deposit metal oxides (SiO_x_, SiN_x_, and Al_2_O_3_) from the gas phases in a vacuum environment to produce a dense layer, which yields an ultra-high gas barrier film [15]. However, these methods are expensive and demanding [7]. Moreover, the recent trend is to produce low-cost barriers and hence common printing and coating techniques are being investigated for the large-scale production [6]. Furthermore, Hauch et al. (2006) reported that for organic solar cells (OSCs) barrier materials having WVTRs of around 10^−3^ g/(m^2^·day^1^) @ 25 °C/40%RH is sufficient for a lifetime of 3–5 years [16]. The production of such barrier films (10^−3^ g/(m^2^·day^1^) @ 25 °C/40%RH) do not require vacuum assisted methods and are within the reach of solution processed techniques [17]. For this purpose, Perhydropolysilazane is being extensively reported in the research as an alternative route for the production of homogeneous, dense, and defect-free silica films from polymeric precursors using simple and economical solution processing techniques [18,19]. A polymer called Perhydropolysilazanes (PHPS) is an inorganic material that consists of silicon and nitrogen atoms in its backbone (-SiH_2_–NH-) and is being used frequently in recent times to produce silica coatings [20,21]. In favorable conditions, perhydropolysilazane produces a homogenous and dense SiO_2_ structure, which has a basic characteristic of being impermeable to environmental gases especially moisture and oxygen [22,23]. This makes PHPS a unique material that can be used in various applications, such as optoelectronic packaging, OLED displays, and transparent films [24,25,26]. One of the advantages of PHPS based coatings is their lower susceptibility to crack formation and shrinkage. This is because of the increase in molecular weight of PHPS during its conversion to silica. The increase in weight is due to a reaction with air and moisture [27,28], which results in volume expansion. In contrast, water or alcohol is released in the sol-gel process, which causes a reduction in molecular weight and consequently, shrinkage occurs [28]. In 1964, Krüger and Rochow [29] introduced the concept of Polysilazanes. In their work, a reaction of chlorosilanes with ammonia was performed which produced tetrameric cyclosilazanes. Further, cyclosilazanes in presence of a catalyst were treated at elevated temperatures. This treatment yielded a polysilazane with high molecular weight. Completely cured polysilazane remains optically clear and transparent yielding a very smooth surface [30,31,32,33]. For complete transformation of polysilazane to silica, different methods are being used that include: curing with heat, exposing to a catalyst, curing by irradiating with deep UV and a combination of the aforementioned methods [24,33].

In the literature, various types of deposition techniques along with curing methods, and strategies have been reported to improve the barrier characteristics of Polysilazane derived coatings [6,17,28,34,35,36]. It is also reported in the literature that the characteristics of Polysilazane (such as permeability against oxygen and moisture) are heavily dependent on the curing process, film thickness, number of layers in a stack, and presence of defects [7,20,37]. Prager et al. produced a single layer of PHPS on PET substrate having a thickness of 250 nm and reduced the permeation of oxygen by a factor of 400 as compared to bare PET. In this work, the PHPS layer was treated with deep UV in a controlled atmosphere containing only 100 ppm of oxygen [18,19] whereas Channa et al., reported almost the same permeation reduction factor with several thin PHPS films treated with deep UV in ambient conditions [7]. Ohishi et al. [34] deposited a thin PHPS layer on a PET-coated ITO substrate and cured it via thermal curing. Deposited PHPS was heated at 140 °C for around 20 min while maintaining a relative humidity level of 90%. In this way, oxygen and moisture permeation of 0.02 cm^3^/(m^2^⋅day⋅bar) and 0.15 g/(m^2^·day) were achieved, respectively. Similarly, Kobayashi et al. [34] studied PHPS films treated by deep UV irradiation under different conditions. It was observed that the films treated with a shorter distance in a controlled oxygen level exhibited WVTR values lower than 0.1 g/(m^2^·day^1^). This indicated irradiation with deep UV at a shorter distance plays a vital role in PHPS bond dissociation and the formation of silica. Ohishi et al. [33] also reported that deep UV irradiated films along with simultaneous heating at 150 °C produced silica that exhibited excellent moisture barrier characteristics, i.e., less than 10^−2^ g /(m^2^⋅day). Ohishi et al. concluded that the reaction rate of deep UV curing is enhanced considerably by raising the substrate temperatures. In another study, PHPS thickness was varied, and the films were treated with a low-pressure mercury lamp (HgLP) having average radiation wavelength of 185 nm. The irradiation was carried out in nitrogen environment while maintaining a low oxygen level [35]. It was observed that the transformation of PHPS to silica not only depend on the curing method, but also on the film thickness. The thick films are hard to transform in silica as compared to thin films. This is because treatment with deep UV causes the surface to transform first, and then with time the transformation proceeds to depth of the layer. The surface that is already transformed into silica acts as the barrier and makes it harder for the oxygen molecules to diffuse through it and hence un-transformed PHPS is left underneath. This transformed PHPS offers path to permeating molecules, which ultimately results in poorer barrier properties. In order to deal with this problem, Morlier et al. [36], created multilayered stack and applied it as the encapsulation of organic solar cells. In this study, a stack of five barrier layers was deposited on a PET substrate. The stack contained one PVA layer sandwiched between two PHPS layers on each side, i.e., PET/PHPS/PHPS/PVA/PHPS/PHPS. This multilayered stack was laminated on a P3HT:PCBM based organic solar cell device. The encapsulation performance in terms of protection of the device was measured and compared with bare PET and a commercial barrier. It was reported that the devices encapsulated with PET degraded first. In contrast, the devices that were encapsulated with the PHPS multilayer stack and commercial barrier showed almost no degradation over the observation period of around 250 h. This suggested that PHPS based films have a high potential to be used as coated encapsulating films for the organic solar cells [36]. Owing to this idea, Channa et al. [17] packaged the devices by direct deposition of PHPS solution and the deposited PHPS was cured with the simultaneous deep UV irradiation and heating at 100 °C. As a result, an increase of the device lifetime in acceleration conditions (40 °C & 85% RH) was observed.

Many researchers have successfully used PHPS as an encapsulating layer for optoelectronic devices, as PHPS produces an excellent barrier against oxygen and moisture. Most of them used different processing strategies and curing methods for imparting the barrier characteristics in the films. In this work, PHPS is coated via a blade coating method and the process parameters are discussed in detail and optimized in terms of curing method, curing environment, and thickness so that a common platform can be created for the researchers who work on the processing of polysilazanes. FTIR is used as the main tool to optimize the curing method and various thin film controlling parameters are discussed to control the final PHPS film characteristics.

## 2. Experimental Section

### 2.1. Materials

Perhydropolysilazane (PHPS NN 120-20) diluted in di-n-butyl ether was received from AZ Electronic Materials GmbH, Wiesbaden, Germany. PET Melinex ST504 with a thickness of 125 µm was obtained from DuPont Teijin Films (Chester, PA, USA) and was used as a substrate after proper cleaning. Commercially used low density polyethylene was obtained from local market and were also used as the substrate. The films were deposited by blade coating (ZAA 2300, manufactured by Zehntner Testing Instruments, Sissach, Switzerland).

### 2.2. Preparation of the Barrier Coatings

Before use, polyethylene/PET substrates were cleaned ultrasonically in acetone and isopropanol bath for five minutes each. The polysilazane was deposited on substrates by blade using different blade gap, pipette volume and coating speeds. After deposition, films were annealed at 50 °C on a hotplate for about 1–2 min to remove the solvent. Then, the coatings were treated with different methods, including damp heat, dry heat, deep UV irradiation and simultaneous deep UV irradiation and heating. For deep UV irradiation an OSRAM XERADEX VUV (OSRAM GmbH, Munich, Germany) source with peak wavelength 172 nm was used. The distance between the surface of the deep UV lamp and the sample was varied between 5–50 mm. For creating multilayers, the same procedure was repeated for each layer.

### 2.3. Characterisation of Films

#### 2.3.1. Spectroscopic Analysis

Transparency of films was analyzed by using a UV-vis device (Shimadzu UV-1800 spectrophotometer, Shimadzu Deutschland GmbH, Duisburg, Germany). IR spectra were recorded in ATR mode with a Fourier transform infrared (FTIR) spectrophotometer (Bruker ALPHA-P, Karlsruhe, Germany) FTIR operating with OPUS 7.2 software. Spectra were obtained using 64 scan summations at 4 cm^−1^ resolutions. Thickness of the samples were measured via a surface profilometer. Contact angle was measured by contact angle goniometer SL200A manufactured by KINO Scientific Instrument, Boston, MA, USA.

#### 2.3.2. Permeability Measurements

Moisture permeability measurements were performed by using 68-3000 EZ-Cup Vapometermanufactured by Thwing Albert, (West Berlin, NJ, USA) complying ASTM E96 for the films having WVTR of more than 1 g/(m^2^.day). Rest of the films were measured by using a moisture permeation analyzer M7002 manufactured by SYSTECH Illinois, Thame, UK. This system has a lower detection limit of 0.02 g/(m^2^⋅day) or 0.002 g/(m^2^⋅day), which depend on the size of the sample.

## 3. Results and Discussion

### 3.1. Various Treatment Methods for Curing Polysilazanes

PHPS layers were coated on polyethylene/PET substrates via doctor blading with applicator gap set at 50 µm and coating speed of 5 mm·s^−1^. Various methods of curing were adopted. These treatment methods for understanding purpose are termed as dry heat curing, damp heat curing and deep UV curing methods. Dry heat curing method includes heating of PHPS coated films at constant 100 °C temperature. In damp heat curing method, the films were exposed to pre-defined condition of 65 °C/85% relative humidity. In deep UV curing method, the films were irradiated with UV light (with wavelength of ~172 nm). To check the effect of each method, the films were analyzed by FTIR spectroscopy.

**Figure 1:**
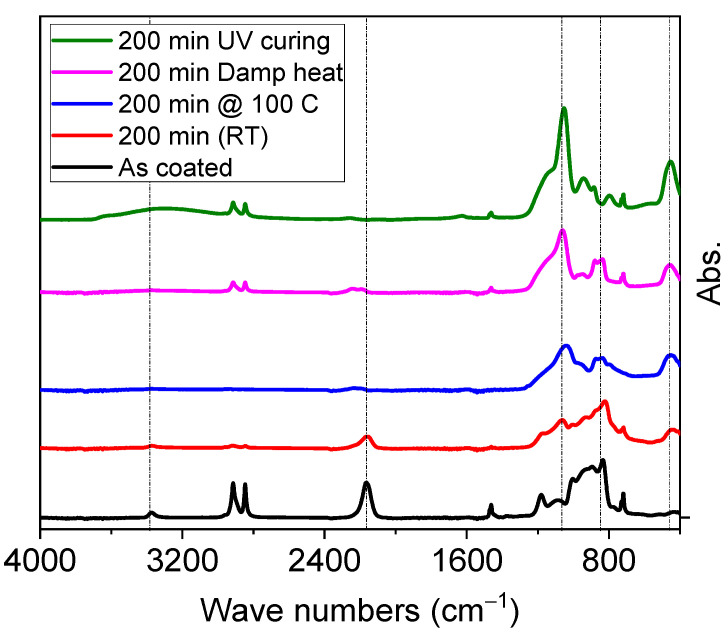
FTIR spectra of PHPS cured with different methods.

Since PHPS reacts with the atmospheric moisture to yield Silica, PHPS was heated at 100 °C, and the conversion was monitored with FTIR. The PET substrate can hold a maximum of 120 °C without showing any warpage, hence, 100 °C is selected. For comparison purposes, PHPS films were also cured at room temperature (~25 °C) in ambient conditions. Figure 1 shows the IR spectra of untreated and treated PHPS films. In agreement with the literature [17,25,28], untreated films (black curve) show peaks at 830 cm^−1^, 2150 cm^−1^ and 3400 cm^−1^, which correspond to Si–N, Si–H, and N–H stretching vibrations, respectively. Peaks around 750, 1180, 1350, 2850, and 2900 cm^−1^ belong to substrate. Red curve shows the IR spectra of PHPS films treated with dry heat at 100 °C (red curve). It can be observed that the peaks near 3400 cm^−1^ (N–H) and 2170 cm^−1^ (Si–H) are nearly gone after 120 min, and at the same time, absorption peaks based on a siloxane bond (-Si–O–Si-) near 450 cm^−1^ and 1050 cm^−1^ appeared and increased. These peaks indicate the transformation of PHPS into SiO_2_. The spectrum for the exposure time 200 min showed absorption peak ratios of *I*(1050 cm^−1^)/*I*(830 cm^−1^) and *I*(450 cm^−1^)/*I*(830 cm^−1^) for black, red, blue, pink and green curves. Room temperature curing (Si-N-Si) indicated that Polysilazane still existed and that the transformation to SiO_2_ was still incomplete. The peak intensities of characteristic peaks *I*(450 cm^−1^), *I*(830 cm^−1^) and *I*(1050 cm^−1^) were recorded to be 0.3096, 0.2786, 0.3857, respectively, which correspond to peak ratios of 1.1114 for *I*(450 cm^−1^)/*I*(830 cm^−1^) and 1.3843 for *I*(1050 cm^−1^)/*I*(830 cm^−1^). A similar but slow transformation mechanism is observed for the films that were left over at room temperature for curing in ambient conditions. Peak intensity ratios of characteristic peaks were recorded to be 0.4120 for *I*(450 cm^−1^)/*I*(830 cm^−1^) and 0.5870 for *I*(1050 cm^−1^)/*I*(830 cm^−1^), which clearly indicates that the curing of PHPS is possible at room temperature, but can take longer times. However, at these conditions, the driving forces for carrying out a reaction between PHPS and atmospheric moisture is very weak. The pink curve refers to the IR spectra of PHPS film cured with controlled damp heat (65 °C/85% relative humidity).

**Figure 2:**
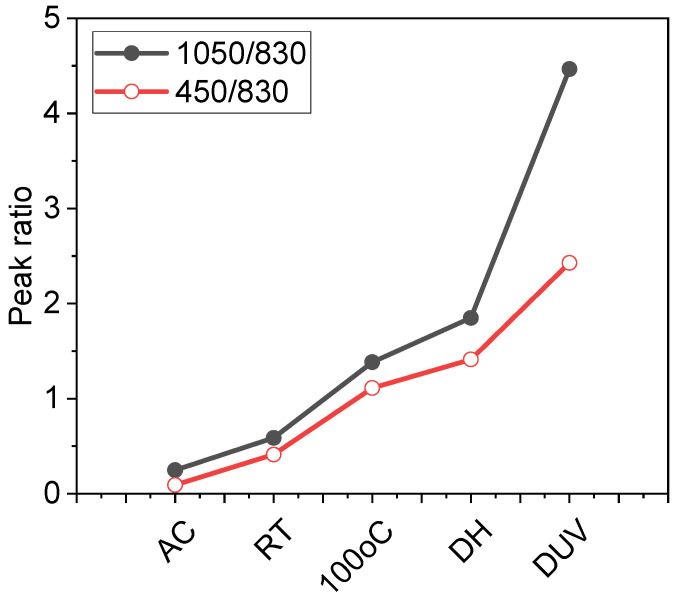
FTIR spectra of PHPS cured with different methods, Black curve with closed black circle represents peak ratio of *I*(1050 cm^−1^)/*I*(830 cm^−1^) for all methods and red curve with open circle represent peak ratio of *I*(450 cm^−1^)/*I*(830 cm^−1^) peaks for all methods. AC, RT, 100 °C, DH and DUV represents as coated, room temperature, heated at 100 °C, damp heat and deep UV, respectively.

The peak intensity ratios clearly describe it as the faster transformation method as compared to dry heat method. This is because the driving force is high enough and the film has lot of moisture molecules to react with, leading to rapid completion of transformation. However, the untransformed PHPS *I*(830 cm^−1^) still remains intact, indicating that the transformation is still not complete. The characteristic peak ratio of *I*(1050 cm^−1^)/*I*(830 cm^−1^) of the films cured with deep UV irradiation was calculated to be 4.46. This peak ratio value is the highest among all peak ratios obtained by other methods. This suggests that almost all the PHPS has been transformed to Silica, and no parent PHPS remains in the film. Out of all methods, deep UV method seems ideal method for the complete transformation of PHPS into silica. For comparison purpose peak ratios of all methods are plotted in Figure 2. This clearly puts deep UV method on top and also in agreement with the literature [17]. However, transformation time of 120 min may be a limiting factor for application of PHPS to large-scale production of barrier layer, where curing time is only few minutes. Therefore, different strategies were applied to reduce this transformation time of PHPS.

#### 3.1.1. PHPS Treatment with Deep UV under Different Environments

**Figure 3:**
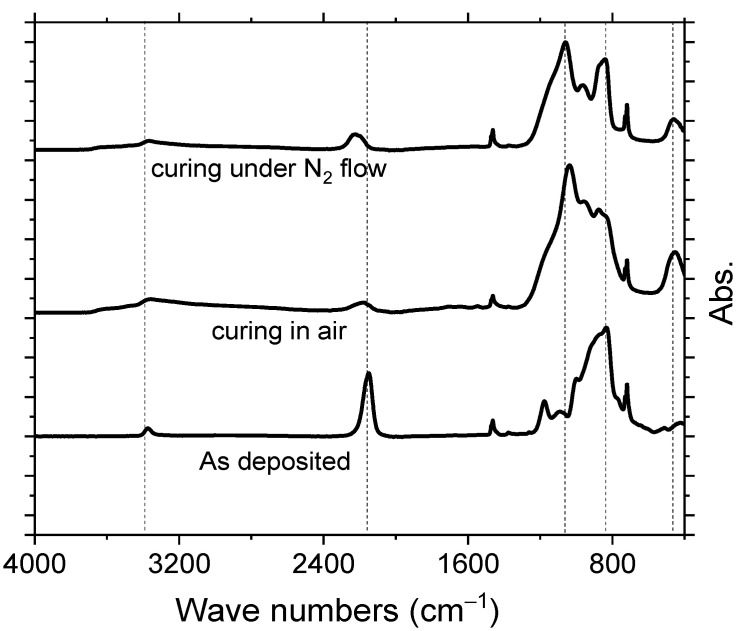
FTIR spectra of PHPS cured under different curing environments.

Experimental data of previous section clearly describe deep UV irradiation of PHPS as a better and faster treatment method for converting it into silica. Almost all PHPS was converted to silica in 200 min. The deep UV treatment of PHPS was carried out in ambient still air. Due to the fact deep UV creates lots of ozone and the presence of ozone molecules between sample surface and deep UV source, which can block light rays resulting in slower transformation of PHPS into silica. This is the reason Prager et al. [18] and Oishi et al. [37] cured PHPS in a closed proximity with predefined oxygen amount. In both cases, the level of oxygen was controlled and maintained at about 100 ppm in a predefined confined area. Here, the point to be noted is treatment of PHPS under closed proximity may not be preferred for large scale production, hence an alternative must be investigated. Hence, PHPS is cured in ambient temperature with constant flow of nitrogen gas and air between sample surface and deep UV source. The purpose was to blow away ozone molecules so that the maximum light ray can reach PHPS surface. The progress of the treatment is monitored by FTIR and results are shown in Figure 3.

PHPS films were irradiated with deep UV, from a distance of 100 mm for 60 min with a constant but milder flow of nitrogen gas and air between sample and deep UV source. Peak intensity ratios of characteristic peaks were recorded to compare the transformation efficiency in all three conditions. *I*(450 cm^−1^)/I(830 cm^−1^) and *I*(1050 cm^−1^)/*I*(830 cm^−1^) for air cured sample was much higher compared to that of nitrogen flown. *I*(450 cm^−1^)/*I*(830 cm^−1^) for air-cured sample is 0.64 in comparison to 0.35 for nitrogen cured sample. Similarly, *I*(1050 cm^−1^)/*I*(830 cm^−1^) for air-cured sample is 1.5 in comparison to 1.22 for nitrogen cured sample. This concludes that the treatment of PHPS under air flow is better than the nitrogen flow, the reason could be the flowing nitrogen blows away not only ozone molecules, but also most of the oxygen radicals, leaving behind only few oxygen molecules that react with PHPS surface. Hence, the transformation of the PHPS into silica is slower, and with constant air flow, the deep UV light can generate enough oxygen atoms that are sufficient to transform PHPS into silica, the flow of air is also favorable in a way that it also blows away ozone which may have hindered the transformation by blocking UV light.

#### 3.1.2. Optimizing the Distance for Faster Transformation of PHPS into Silica

**Figure 4:**
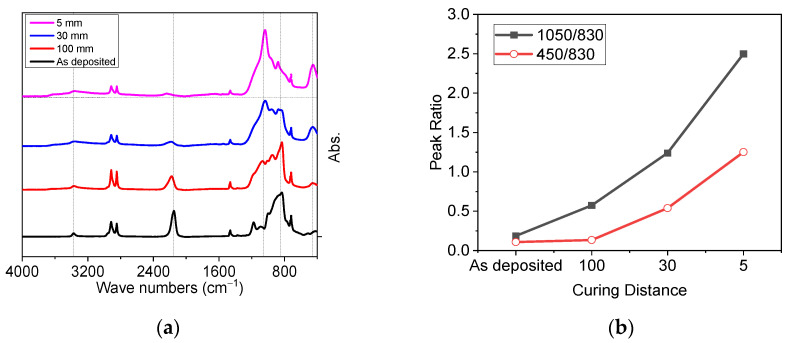
FTIR spectra of PHPS and corresponding peak ratios. (**a**) FTIR spectra of PHPS cured with different curing distances, (**b**) peak ratios of peak where black curve with closed squares represents peak ratio of *I*(1050 cm^−1^)/*I*(830 cm^−1^) and red curve with open circle represents *I*(450 cm^−1^)/*I*(830 cm^−1^) peak intensity.

To further reduce the transformation time, the distance between deep UV source and sample surface was varied. In all previous experiments, the distance between source and sample was kept 100 mm. In this section the distance is step by step reduced and the irradiation time is kept 20 min. During the irradiation, a gentle but constant air flow was maintained. The variation of the irradiation distance is 100, 30, and 5 mm. FTIR spectra of treatment PHPS films are shown in Figure 4a, and corresponding peak ratios are given in Figure 4b.

The enhanced transformation of curing samples was achieved by decreasing the distance between the UV source and the sample surface. The best transformation is achieved at 5 mm distance, represented by highest values of *I*(450 cm^−1^)/*I*(830 cm^−1^) and *I*(1050 cm^−1^)/*I*(830 cm^−1^) (i.e., 1.25 and 2.5, respectively). On the other hand *I*(450 cm^−1^)/*I*(830 cm^−1^) and *I*(1050 cm^−1^)/*I*(830 cm^−1^) were found to decrease with increased distance. It can be concluded that the treatment of PHPS at smallest distance is favorable. As at this distance most of the light reaches to PHPS surface and gentle flow kicks away the ozone molecules and hence the transformation of PHPS proceeds to completion at faster pace.

#### 3.1.3. Fastest Curing

**Figure 5:**
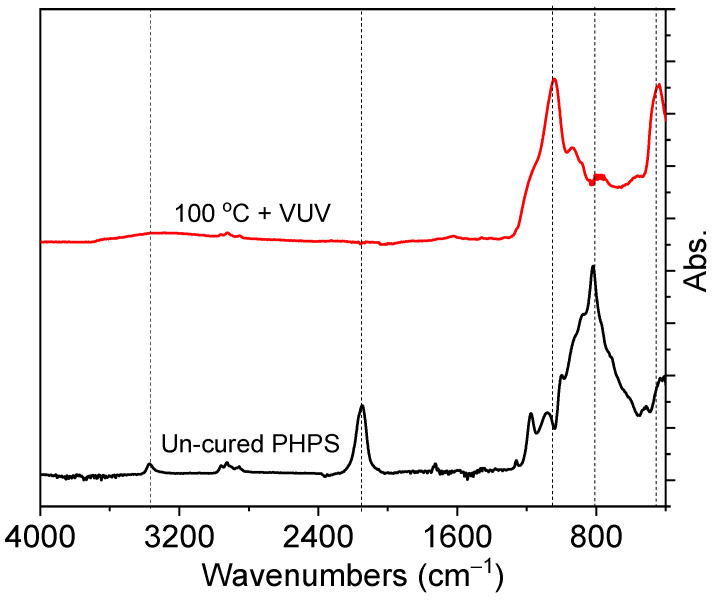
FTIR spectra of PHPS cured with combination of heat and simultaneous irradiation with deep UV at a distance of 5 mm and compared with un-cured PHPS.

Ohishi et al. [37] showed that the fastest method to cure PHPS is the combination of the above methods. This is because, while curing PHPS forms a good barrier on top, it then becomes difficult for oxygen molecules to penetrate and react. Therefore, additional heat causes them to penetrate and form SiO_2_ network and hence it is the quickest method. In this section the same strategy is applied to PHPS films at smaller distance, i.e., 5 mm with gentle but constant air flow and results in terms of FTIR spectra are shown in Figure 5. The best performance was achieved. The highest values for *I*(450 cm^−1^)/*I*(830 cm^−1^) and *I*(1050 cm^−1^)/*I*(830 cm^−1^) (i.e., 2.471 and 2.553, respectively) were received for the samples cured in deep UV irradiation with heat. This means most of the PHPS has been converted and much less parent PHPS remains in the layer by using combination of deep UV irradiation with heat at a lower distance with constant air flow. This implies the best way to cure PHPS films is the combination of deep UV irradiation with heat at lower distance with constant air flow. Deep UV irradiation creates oxygen molecules and heat causes them to diffuse deeper in the layer and react with PHPS, and constant flow of air blows the ozone away. Due to this, PHPS is converted into silica at a rapid pace. The conversion time of ~5 min is well suited to many applications including large scale roll-to-roll manufacturing of gas barriers, which may open new and vast applications for PHPS.

### 3.2. Transparency and Contact Angle Measurements of PHPS

**Figure 6:**
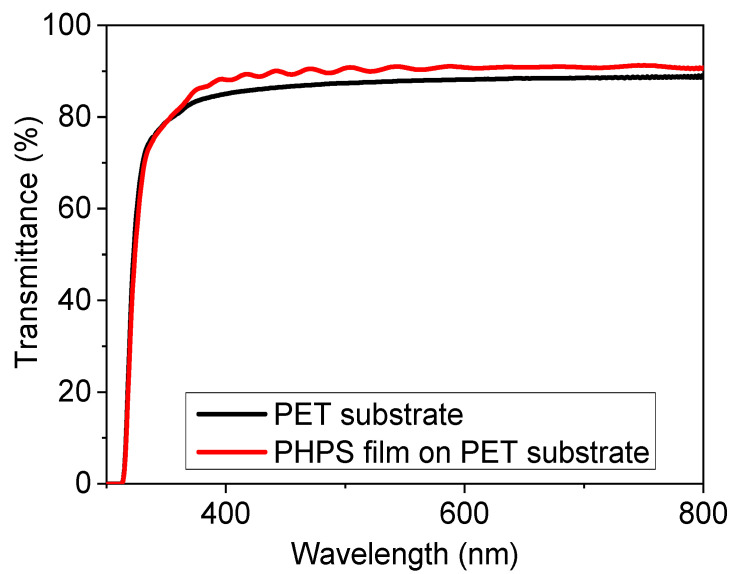
UV vis transmittance spectra of 3 layers of PHPS (each having thickness of ~700 nm) stacked on top of each other.

A stack of PHPS films (3 layers) on PET substrate each PHPS layer was treated with the fastest curing method, i.e., combination of heat and deep UV irradiation using 5 mm distance; were subjected to transparency test and result is shown in Figure 6.

**Figure 7:**
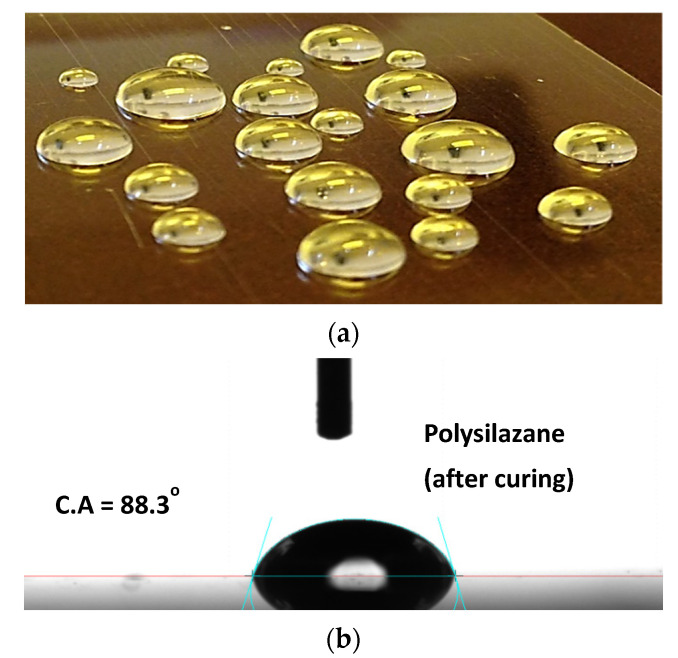
Surface properties of PHPS cured films, (**a**) shows a photo of droplets and their structure on PHPS films coated on PET substrate, and (**b**) shows the exact contact angle measured on water droplet formed on PHPS cured film.

The PET substrate showed a transparency of around 88% in the visible region and the three stacked coating of PHPS on PET showed a transparency of around 91%. The fluctuations in the range of 280 to 500 nm could be due to the interference effect of the three stacked layers as reported in the literature (21). The reason for the slight (3%) increase in transmittance is that the PET substrate is shiny and reflects light and PHPS coating reduced its reflectance and, as a result, transmittance increased. This result also suggested that the PHPS coatings can also be used as an anti-reflecting coating. Apart from this, PHPS cured films also showed a slight hydrophobic nature as the contact angle measurements were recorded to be around 91° as shown in Figure 7a,b. Figure 7a shows the structure of the droplets on the PHPS coated substrate, and Figure 7b shows the contact angle measurement.

### 3.3. Control of PHPS Film Thickness

In order to get control over the thickness of the cured PHPS films, various parameters were tested. All tested parameters are shown in Table 1.

The as received PHPS solution was in 20 wt% concentration in di-butyl ether solvent. The film prepared via doctor blading from the as received solution yields a thick (~800 nm) film. In order to get much thinner films, the PHPS solution was diluted with di-butyl ether solvent in different ratios and processed via doctor blading with various controlled parameters such as blade gap and coating speed. Wet layer thickness for all films was set to 400 µm on the PET substrate, and cured with combination of deep UV and heating at 100 °C at a curing distance of 5 mm. It was found that the thinnest PHPS cured film was obtained when the PHPS was diluted in di-butyl ether at a dilution ratio of 1:6, that means one part of PHPS original solution and 6 parts of di-butyl ether solvent mixture and processed with the lowest possible coating speed. Thicker films around 1 um and thinnest films around 70 nm both are possible with PHPS, and obtaining control over these processing parameters provides full control to the processor to get a desired film thickness.

#### Barrier Properties

Historically, SiO_2_ has been an excellent barrier against the diffusion of various gases. To check the quality of the SiO_2_ formed from PHPS solution, barrier characteristics of films were measured in terms of oxygen and moisture.

**Figure 8:**
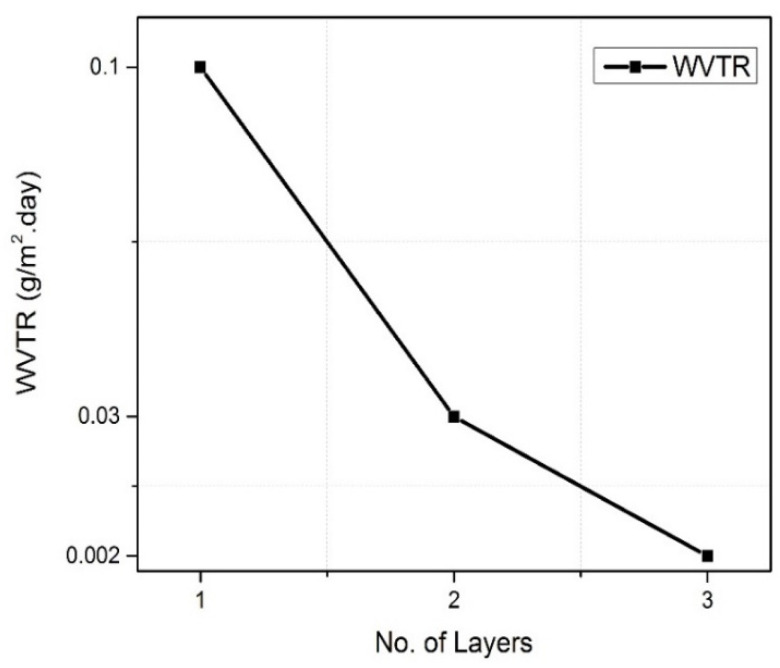
Barrier properties of multilayers of PHPS coated on PET substrate, each layer cured with simultaneous deep UV irradiation and heat.

The PHPS films cured with the different curing methods, such as room temperature curing under ambient conditions, heating at 100 °C in ambient relative humidity, controlled damp heat conditions (65 °C/85%RH), irradiation with deep UV Light and simultaneous deep UV irradiation and heating at 100 °C, are evaluated for their permeation against moisture. It was found that the films cured at room temperature show very rough WVTR values (4 g/m^2^·day), which are the same as the barrier property of the PET substrate, which means the room temperature cured PHPS did not form a fully dense layer, which resulted in poor barrier characteristics. The films cured with dry heat at 100 °C in ambient RH%, showed slightly better WVTR values (around 2.2 g/(m^2^⋅day)). The films cured in controlled damp heat conditions showed WVTR values of 1.7 g/m^2^.day, and finally, the films cured with simultaneous deep UV irradiation and heating at 100 °C showed an excellent barrier property <0.1 g/(m^2^⋅day). It can be inferred from the values that the films cured at room temperature in ambient conditions did experience a transformation, but that transformation was not complete, as already discussed in the curing section. The surface of the films may still contain un-transformed PHPS, which acts as the diffusing medium for environmental gases. The best barrier properties were observed when the films were simultaneously irradiated with deep UV and heat. The barrier properties so far were recorded to be 0.01 g/(m^2^⋅day). This barrier value was further reduced to below 0.002 g/(m^2^⋅day) when the three coatings on top of each other were prepared (Figure 8). The reason for this extreme low barrier properties could be the combination of the excellent formation of each PHPS layer into SiO_2_ and simultaneously offering a tortuous path to permeating molecules. A diffusion molecules molecule may have to travel laterally until they meet the defect or un-transformed PHPS to reach the other side, and hence low permeation rates are recorded [23].

## 4. Conclusions

In this work, the optimization of the PHPS processing is performed in terms of curing method including treating the PHPS with room temperature, dry heating at 100 °C, irradiation with deep UV, exposing the films to damp heat in controlled conditions of 65 °C temperature along with fixed relative humidity level of 85% and simultaneous deep UV irradiation with substrate temperature maintained at 100 °C. It was optimized so that the PHPS films can be transformed to silica completely in less than 5 min when cured by deep UV irradiation from a lower distance and maintaining a substrate temperature of 100 °C. This method is around 40 times faster than the curing with deep UV irradiation in ambient conditions. The cured films exhibited the transparency of about 92% in the white light region and maintained the slight hydrophobic nature as that of the parent PHPS. Furthermore, various parameters were tested to optimize the film thickness, and it was found that the dilution ratio along with film coating speed plays a vital role in final film thickness. A wide range of film thickness from a few nanometers to a micrometer is demonstrated with slight variation of dilution and coating speed. Creating multilayers of PHPS over top each other is also favorable, and they exhibit a water vapor transmission rate of less than 0.002 g/(m^2^·day). The obtained barrier films fully satisfy the requirements of the encapsulation of the organic photovoltaic devices in all terms, including barrier values, transparency, as well as processing. Moreover, these barrier films are completely processed in ambient conditions, which is a clear advantage for the large-scale barrier production at a reasonably low cost.

## Figures and Tables

**Table 1:** Parameters for coating PHPS layers on PET substrate.

Material (Amount) (70 µL)	Coating Speed (mm/s)	Dilution (ratio)	Final Thickness (nm)	Curing Time (min)
PHPS	1 mm/s	1:1	~450 nm	10
PHPS	5 mm/s	1:1	~700 nm	10
PHPS	10 mm/s	1:1	~1200 nm	15
PHPS	15 mm/s	1:1	~1500 nm	20
PHPS	20 mm/s	1:1	~1600 nm	20
PHPS	30 mm/s	1:1	~2500 nm	20
PHPS	1 mm/s	1:6	~70 nm	2–3
PHPS	1 mm/s	1:5	~100	2–3

## Data Availability

Data available upon request from the corresponding author(s).
